# Association of ADHD and Obesity in Hispanic Children on the US-Mexico Border: A Retrospective Analysis

**DOI:** 10.3389/fnint.2021.749907

**Published:** 2022-01-05

**Authors:** Alyssa Salcido, Eden Hernandez Robles, Kiran Chaudhary, Luis Alvarado, Sergio D. Iñiguez, Javier Vargas-Medrano, Valeria Diaz-Pacheco, Maria Theresa Villanos, Bharathi S. Gadad, Sarah L. Martin

**Affiliations:** ^1^Department of Psychiatry, Paul L Foster School of Medicine, Texas Tech University Health Sciences Center El Paso, El Paso, TX, United States; ^2^Biostatistics and Epidemiology Consulting Lab, Texas Tech University Health Sciences Center El Paso, El Paso, TX, United States; ^3^Department of Psychology, The University of Texas at El Paso, El Paso, TX, United States; ^4^Department of Psychiatry, Texas Tech University Health Sciences Center, El Paso, TX, United States; ^5^Department of Pediatrics, Paul L. Foster School of Medicine, Texas Tech University Health Science Center, El Paso, TX, United States

**Keywords:** pediatric obesity, body mass index, attention deficit and hyperactivity disorder (ADHD), Hispanic population, US-Mexico border

## Abstract

Pediatric obesity and Attention Deficit Hyperactivity Disorder (ADHD) are rising health concerns in the United States, especially among Hispanic children and adolescents. Research on Hispanic children and adolescents indicates disproportionately higher prevalence rates of obesity in this community but scant data on ADHD prevalence rates. In contrast, a plethora of research studies across the general population examines the relationship between childhood obesity and ADHD. In addition, there is a lack of research that examines the role of ethnicity and sub-ethnic group correlations in ADHD, particularly in the Hispanic population. Existing studies in the general population indicate ADHD may be a risk factor for being overweight compared to normal controls. The objective of the present study is to examine the prevalence of obesity in children with ADHD compared to children in the general population in a predominately Hispanic sample on the US-Mexico border. A total of 7,270 pediatric medical records were evaluated. The retrospective analysis included Body Mass Index (BMI) and related health variables, and ethnicity and showed that children with ADHD are more likely to be underweight. In conclusion, no significant relationship existed between obesity and ADHD among Hispanic children on the US-Mexico Border, and instead we found the opposite correlation.

## Introduction

Pediatric obesity in the United States is increasing rapidly, with a prevalence rate of about 32% of children and adolescents classified as overweight (Centers for Disease Control and Prevention [CDC], 2020). Obesity rates among Hispanic children are rising at a higher rate than non-Hispanic White children (Isasi et al., 2016), and a variety of mental health conditions are often linked to obesity, although for various reasons (Girela-Serrano et al., 2021; Rojo et al., 2021). Severe obesity is more common in Hispanic children, with reports indicating development as early as kindergarten (Isasi et al., 2016; Kim et al., 2016). Overweight children are twice as likely to exhibit elevated rates of Attention Deficit Hyperactivity Disorder (ADHD) symptoms than their average-weight counterparts. In some studies, children with ADHD are twice as likely to be overweight (Cortese, 2019) therefore, it is a public health priority to examine ADHD and its relationship with obesity in Hispanic children.

Hispanic children are more likely to be impacted by obesity-related chronic diseases such as Type 2 Diabetes due to higher insulin resistance and appearance of pre-diabetes than non-Hispanic White children (Soltero et al., 2017). Pediatric obesity is known to mediate detrimental complications later in life, such as diabetes, hypertension, cardiovascular, musculoskeletal, and gastrointestinal issues (Collins and Cleary, 2016; Centers for Disease Control and Prevention [CDC], 2020). Children not only suffer from physical complications of obesity, but they also may suffer from psychosocial complications. The psychosocial issues related to obesity include negative self-body image, bullying, weight-related discrimination and victimization in peer interactions, and negative school experiences (Small and Aplasca, 2016).

Extensive research has documented the impact of health inequality factors, such as lower socioeconomic status (SES) and lack of health insurance, on childhood obesity for Hispanic children. When including migration status, Hispanic children with a low SES are at a greater risk for developing negative health behavior patterns and higher BMI scores (Jacques-Tiura and Greenwald, 2016). In addition, the cost and accessibility of healthy foods, unsafe neighborhoods, and preventive medical care access issues negatively impact obesity rates for Hispanic children (Lutfiyya et al., 2008).

Increased obesity rates are also prevalent in children with psychiatric disorders such as ADHD, the most common childhood neurodevelopmental disorder (Small and Aplasca, 2016). Studies have shown a connection between ADHD and obesity, with ADHD as a risk factor for being overweight compared to normal controls (Cortese et al., 2008; Lutfiyya et al., 2008). A systematic review and meta-analysis by Nigg et al. (2016) indicated significant associations in females compared to males and larger associations in adults compared to children (Nigg et al., 2016). Clinical studies often report mixed results on the association between ADHD and obesity. Still, systematic reviews and meta-analyses of population-based and clinical studies indicated an association between ADHD and obesity. A significant association between ADHD and obesity was found for children, although at a lesser effect than adults (Cortese et al., 2008, 2016; Cortese and Tessari, 2017; Cortese, 2019). Of note, the country where the study was conducted did not significantly impact the association between obesity and ADHD (Cortese et al., 2016; Cortese and Tessari, 2017; Cortese, 2019), but it is unknown whether a unique geographic location, where two countries converge such as the U.S. Mexico Border, will influence the association. The correlation between ADHD and childhood obesity could be due to various factors such as medication, genetic predisposition, and environmental factors (Jacques-Tiura and Greenwald, 2016). Based on the review literature of these studies, it is critical to investigate the ADHD and obesity prevalence among a predominantly Hispanic sample from the US-Mexico Border. Therefore, the goals of this study are (1) to determine the prevalence of obesity in children with ADHD compared to children in the general population in a predominately Hispanic community on the US-Mexico Border and (2) to determine if socioeconomic factors affect BMI in this population, in order to inform plans for future health interventions to reduce obesity and treat ADHD in children of Hispanic descent.

## Materials and Methods

The Texas Tech University Health Sciences Center at El Paso operates as a medical teaching healthcare system on the US-Mexico Border. El Paso is a US-Mexico border community with a predominantly Hispanic population and is within close proximity to Ciudad Juarez a major metropolitan city of Mexico. The community is a particularly unique population in that both Mexican-born and US-born, individuals travel back and forth. While we do not have data on the patients’ migration circulatory patterns, we understand that the population is cross-border mobile.

We performed a retrospective analysis of electronic health records of pediatric patients receiving services from one of two clinics – our Child and Adolescent Psychiatry clinic and our Geneneral Pediatrics clinic. Patient records were included if the patient was between 6–17 years of age on the date of the visit. Patients records were considered in the analysis if the patient was diagnosed with ADHD meeting Diagnostic and Statistical Manual of Mental Disorders V (DSM-5) criteria. Only the most recent patient visit was considered for analysis in the study.

Body mass index calculations were determined using the weight and height at the time of the visit and included age and sex-specific differences. The calculation for BMI was determined using the Center for Disease Control and Prevention (CDC) guidelines for pediatric patients. As a part of routine care, height, weight, age, and sex-specific information is obtained at the time of the visit. Pediatric BMI categories were separated into the following categories: (1) Underweight (<5th percentile); (2) Normal or Healthy Weight (5–85th percentile); Overweight (85–95th percentile); or Obese (>95th percentile).

Pediatric patient records selected for data analysis included controls (without a diagnosis of ADHD at any time point) and pediatric patients diagnosed with ADHD (at any time point). Patients were diagnosed with ADHD through (a) a comprehensive developmental history, (b) clinician interview, and (c) direct observation. Patient records were sorted into one of the four subtypes of ADHD. The four subsets included: (1) ADHD-combined type, (2) ADHD-unspecified type, (3) ADHD-predominantly inattentive type, and (4) ADHD-predominantly hyperactive/other types. In addition, we considered any patients diagnosed with ADHD at any point for analysis in the ADHD group.

Furthermore, we analyzed patients by ethnicity and language. The definitions for ethnicity and language were pre-determined using Medicaid/Medicare measures, and direct data collection methods were used for this information. For analysis purposes, patients were categorized into traditional general categories by ethnicity – Hispanic or Latino and Not Hispanic or Latino, and language – English or Spanish or Other/missing. The Institutional Review Board (IRB) approved the study. Personal protected information in data collection was de-identified.

Key descriptive variables (age, BMI, gender, ethnicity, language, diagnosis status, insurance status, and diagnosis subset) were summarized. Association and relationships between the variables were analyzed using an unpaired *t*-test and Fisher’s Exact test depending on the data distribution. A univariate and multivariable relative risk regression reviewed the risk of ADHD as compared to non-ADHD patients. Relative risk (rr), 95% confidence interval, and *p*-values describe the models. *p*-values were considered statistically significant at 5%. Analysis of the data was performed by using Stata software, SE edition.

## Results

### Demographics

We extracted 7,270 electronic health records of pediatric patients receiving clinic services between 2017–2018 (Table 1). The mean age of the sample was 11.64 years old (SD = 3.46), and the sample is 50.19% male (49.81% female). The majority (92.31%) were Hispanic or Latino, with 7.69% as Not Hispanic or Latino. The majority (71.07%) reported having Medicaid as their primary insurance, whereas 28.93% had private insurance or self-pay. Almost half (59.62%) had a normal BMI, and only 6.24% had an underweight BMI. Thus, less than half (15.18 and 18.96%, respectively) of our sample had a BMI in the 85–95th percentile (overweight) or the 95th percentile (obese). Among individuals diagnosed with ADHD (7.74%), 57.02% were combined type, 21.49% were unspecified type, 17.94% were predominately inattentive type, and 3.55% were predominantly hyperactive or other types (Table 1).

**TABLE 1:** Overall descriptive statistics.

Factor	Value
N	7270
AGE, mean (SD)	11.64 (3.46)
BMI, mean (SD)	20.40 (5.71)
**BMI**	
Underweight (<5th percentile)	430(6.24%)
Normal Weight (5–85th percentile)	4109(59.62%)
Overweight (85–95th percentile)	1046(15.18%)
Obese (≥95th percentile)	1307(18.96%)
**Gender**	
Male	3649(50.19%)
Female	3621(49.81%)
**Ethnicity**	
Not Hispanic or Latino	548(7.69%)
Hispanic or Latino	6578(92.31%)
**Language**	
English	2424(33.34%)
Spanish	2524(34.72%)
Other/missing	2322(31.94%)
**Diagnosis status**	
Non-ADHD	6707(92.26%)
ADHD diagnosis	563(7.74%)
**Insurance status**	
Medicaid/medicare	5036(71.07%)
Private/self pay	2050(28.93%)
**Diagnosis subset**	
ADHD-combined type	321(57.02%)
ADHD-unspecified type	121(21.49%)
ADHD-predom inattentive type	101(17.94%)
ADHD-predom hyperactive/other type	20(3.55%)

### Association Between Attention Deficit Hyperactivity Disorder With Body Mass Index, Gender, Ethnicity, Language, and Insurance Status

Based on the unpaired *T*-Test and Fisher’s exact test, a marginal statistical significance was detected in BMI categories as shown in Table 2. Significant mean differences were also indicated among gender categories. Namely, differences among males (71.40%) and females receiving ADHD diagnoses (28.60%) were detected (*p* < 0.001). Differences were detected among Hispanic or Latino or Not Hispanic or Latino ethnicity diagnosed with ADHD and among those diagnosed with ADHD (*p* < 0.001). Findings for language detected significant mean differences by language by ADHD status (*p* < 0.001) (Table 2).

**TABLE 2:** Association between ADHD status and variables of interest.

Factor	Non-ADHD	ADHD diagnosis	*p*-value
N	6707	563	
AGE, mean (SD)	11.66 (3.49)	11.31 (3.07)	0.022
BMI, mean (SD)	20.47 (5.75)	19.60 (5.17)	0.001
BMI			0.054
Underweight (<5th percentile)	388 (6.07%)	42 (8.40%)	
Normal weight (5–85th percentile)	3801 (59.46%)	308 (61.60%)	
Overweight (85–95th percentile)	984 (15.39%)	62 (12.40%)	
Obese (≥95th percentile)	1219 (19.07%)	88 (17.60%)	
Gender			<0.001
Male	3247 (48.41%)	402 (71.40%)	
Female	3460 (51.59%)	161 (28.60%)	
Ethnicity			<0.001
Not Hispanic or Latino	484 (7.30%)	64 (12.80%)	
Hispanic or Latino	6142 (92.70%)	436 (87.20%)	
Language			<0.001
English	2284 (34.05%)	140 (24.87%)	
Spanish	2422 (36.11%)	102 (18.12%)	
Other/missing	2001 (29.83%)	321 (57.02%)	
Insurance status			<0.001
Medicaid/medicare	4695 (71.81%)	341 (62.23%)	
Private/self pay	1843 (28.19%)	207 (37.77%)	

### Univariate Logistic Regression (Relative Risk) of Attention Deficit Hyperactivity Disorder Status and Body Mass Index

Logistical regression analyses revealed that ADHD status leads to increased risk of being underweight (<5th percentile) and is statistically significant with a *p*-value of 0.047. However, ADHD status showed moderate association with the normal weight category (5–85th percentile, with a *p*-value of *p* < 0.047). Further, the ADHD group had a decreased risk of being in the overweight category (85–95th percentile with a *p*-value <0.442).

The analysis indicated a 60% decreased risk of being female and being diagnosed with ADHD, indicating the risk of ADHD might be more prevalent in males. There is also a 44% lower risk of being Hispanic and being diagnosed with ADHD. Related to insurance type, a 50% increased risk of being diagnosed if a patient has private insurance versus Medicare/Medicaid was detected (Table 3).

**TABLE 3:** Univariate logistic regression (relative risk) of ADHD status.

Variable	RR	95% CI		*p*-value
Age	0.97	0.95	0.99	0.028
BMI (ref – obese)				
Underweight (<5th percentile)	1.45	1.00	2.09	0.047
Normal Weight (5–85th percentile)	1.11	0.87	1.41	0.375
Overweight (85–95th percentile)	0.88	0.64	1.22	0.442
Female	0.40	0.33	0.48	<0.0001
Hispanic or Latino (ref: non-Hispanic)	0.56	0.44	0.74	<0.0001
Spanish (ref – english)	0.69	0.54	0.9	0.006
Private/self pay (ref: medicare/medicaid)	1.49	1.25	1.77	<0.0001

### Association Between Attention Deficit Hyperactivity Disorder Subset Status and Body Mass Index, Gender, Ethnicity, Language, and Insurance Status

Statistical differences were detected among ADHD subset status and age (Table 4). Significant associations are indicated between specific ADHD status (subset) and age. Gender also had a significant association with ADHD sub-set status (*p* < 0.006). No significant associations were detected when accounting for ethnicity, but the language showed a significant association to ADHD subset status (*p* < 0.002). No significant association was found between ADHD subset status and insurance status (*p* > 0.68) (Table 4).

**TABLE 4:** Association between specific ADHD status (subset) and variables of interest.

Factor	ADHD-combined type	ADHD-unspecified type	ADHD-predom inattentive type	ADHD-predom hyperactive/Other type	*p*-value
N	321	121	101	20	
AGE, mean (SD)	10.79 (3.06)	12.12 (2.82)	12.23 (2.93)	10.25 (3.32)	<0.001
BMI, mean (SD)	19.19 (4.69)	19.93 (5.88)	20.69 (5.74)	18.26 (3.31)	0.06
BMI categories					0.99
Underweight (<5th percentile)	24 (8.70%)	11 (9.57%)	6 (6.52%)	1 (5.88%)	
Normal weight (5–85th percentile)	170 (61.59%)	70 (60.87%)	56 (60.87%)	12 (70.59%)	
Overweight (85–95th percentile)	32 (11.59%)	14 (12.17%)	14 (15.22%)	2 (11.76%)	
Obese (≥95th percentile)	50 (18.12%)	20 (17.39%)	16 (17.39%)	2 (11.76%)	
Gender					0.006
Male	242 (75.39%)	89 (73.55%)	59 (58.42%)	12 (60.00%)	
Female	79 (24.61%)	32 (26.45%)	42 (41.58%)	8 (40.00%)	
Ethnicity					0.18
Not Hispanic or Latino	43 (15.36%)	11 (10.48%)	7 (7.29%)	3 (15.79%)	
Hispanic or Latino	237 (84.64%)	94 (89.52%)	89 (92.71%)	16 (84.21%)	
Language					0.002
English	76 (23.68%)	26 (21.49%)	31 (30.69%)	7 (35.00%)	
Spanish	45 (14.02%)	24 (19.83%)	30 (29.70%)	3 (15.00%)	
Other/missing	200 (62.31%)	71 (58.68%)	40 (39.60%)	10 (50.00%)	
Diastolic BP, mean (SD)	62.60 (9.72)	64.98 (8.02)	64.97 (9.32)	66.12 (6.77)	0.023
Systolic BP, mean (SD)	107.94 (11.53)	110.02 (9.48)	108.82 (13.16)	107.71 (11.08)	0.41
Insurance status					0.68
Medicaid/medicare	188 (60.45%)	79 (66.39%)	63 (63.64%)	11 (57.89%)	
Private/self pay	123 (39.55%)	40 (33.61%)	36 (36.36%)	8 (42.11%)	

### Multivariable Logistic Regression (Relative Risk) of Attention Deficit Hyperactivity Disorder Status

When the variables were compared individually with ADHD status, an increased risk of being underweight was identified. Females, Hs, and primary Spanish speakers had a decreased risk of having ADHD. Overall, after considering the quantitative version of BMI and adjusting for gender, ethnicity, and language as the children’s weight increased, there was a lower risk of being diagnosed with ADHD (children diagnosed with ADHD were less likely to be overweight) (Table 5).

**TABLE 5:** Multivariable logistic regression (relative risk) of ADHD status.

Variable	RR	95% CI		*P*-value
BMI	0.98	0.96	0.99	0.037
Female	0.41	0.33	0.51	<0.0001
Hispanic or Latino (ref: non-Hispanic)	0.85	0.63	1.14	0.281
Spanish (ref – english)	0.71	0.54	0.93	0.013

## Discussion

This study shows that Hispanic children with ADHD are more likely to be underweight than overweight. While these results add to the growing body of research on the association between ADHD and obesity that indicates lower effects in children,(Fliers et al., 2013; Kummer et al., 2016; Cortese, 2019) it raises other important questions surrounding the factors that contribute to these marked differences. Most notably, these findings raise important questions on the role of ethnicity, gender, and other social determinants of health. Whereas we anticipated differences in BMI and ADHD status, the lower risk of being overweight in Hispanic children with ADHD in our border region can be explained in various ways. One possible explanation may be that this population is very likely to follow a standard of care treatment plan for ADHD. Treatment plans for patients with ADHD often include treatment with the stimulant class of medication, which have appetite suppression as a very common side effect, and adding exercise to a child’s schedule. If compliance with a recommended treatment plan is higher in this population than in others, then local cultural factors need to be further explored so that it can be replicated in other communities, leading to lower rates of obesity. Since there are many other studies that show adults with ADHD are more likely than children with ADHD to be overweight and obsese, replicating these factors in adult populations would be very important to the overall health of adult populations.

While the focus of the study was to examine the relationship between ADHD diagnosis and BMI, the decreased risk of an ADHD diagnosis associated with being of Hispanic ethnicity. The present findings are consistent with other population-based studies that indicate lower rates of ADHD diagnosis among Hispanic children compared to non-Hispanic children (Rothe, 2005; Slobodin and Masalha, 2020). However, the reasons behind these findings remain elusive, and may lay in genetics, but may be related to cultural beliefs of both parents and medical providers. Parents identifiying as Hispanic or Latino may be more defended against offering information to a provider about the possible psychiatric problems of their children. Medical providers may have biases toward this population that result in fewer diagnoses of ADHD, even though the disorder may be present in numbers equal to White children. Of course, both of these factors may be simultaneously influencing the likelihood of giving a diagnosis of ADHD.

Related to the present study, the methods for diagnosing patients with ADHD are through clinical interview with the patients and parents or guardians and the reporting of symptoms. Perhaps, during those provider-patient interactions, parents or guardians attitudes and beliefs influenced the reporting of symptoms related to mental illness. Prior studies conducted by Gallo et al. (2009) indicated cultural differences in beliefs surrounding mental illness and mental health treatment (Gallo et al., 2009), and another study by Eiraldi and Diaz (2010) indicated Hispanic parents’ reluctance to accept medication for mental health treatment for their children (Eiraldi and Diaz, 2010). However, these ethnic differences in ADHD diagnosis are due to the protective factors related to culture (Maloney, 2010; Fliers et al., 2013). In the present study, linguistic feature, often a proxy for culture and cultural differences, is also associated with a decreased risk of ADHD diagnosis. The study samples collected are from the US-Mexico border, hence might have certain cultural protective factors that shape the environments of the patients and greater cultural capital. Nevertheless, the findings of the present retrospective analysis provide an opportunity for further exploration of the ethnicity differences in ADHD diagnosis.

An interesting result from this retrospective analysis is the finding of increased risk of being diagnosed with ADHD if using private insurance compared to those with Medicaid-related insurance. This is also found in other studies (Slobodin and Masalha, 2020). The reason for this correlation remains unclear, but the possible explanation is that increased access to educational resources related to mental illness or mental health treatment among those with private insurance accounts for this finding. A plausible interpretation is that with greater income, the ability to attain private insurance increases and access to educational resources that improve understanding of mental illness and mental health treatment also increases, which leads to parents and guardians identifying these symptoms more often. Perhaps a further investigation into factors associated with socioecomic status (SES) and environmental factors related to SES status could provide a better understanding.

There are important limitations to the present retrospective analysis. Primarily, this study relies on less rigorous research methods for diagnosing ADHD than a prospective study. The means for calculating BMI are standardized, yet the methods for arriving at ADHD diagnosis are more variable. Considering our findings on Hispanic ethnicity and ADHD diagnosis and BMI status, interpreting the factors behind these findings would require greater exploration using prospective techniques.

Finally, there are limited studies that have examined ADHD and obesity in children in predominantly Hispanic populations, even fewer that explore unique geographic locations where two countries converge, such as this region on the US-Mexico border. Patients with ADHD are less likely to be overweight and obese than those without ADHD. As obesity continues to be a prevalent health problem in American children, it is important to continue finding possible interventions to decrease the rate of obesity in the pediatric population. Further investigations into understanding what factors contribute to these increased rates of ADHD based on ethnicity and insurance status must be conducted to improve diagnosis, treatment and the overall health of communities.

## Data Availability Statement

The original contributions presented in the study are included in the article/supplementary material, further inquiries can be directed to the corresponding authors.

## Ethics Statement

The research protocol has been reviewed by the Institutional Review Board (IRB) at TTUHSC-El Paso. The IRB Committee, Texas Tech University Health Sciences Center, El Paso have reviewed and approved the research protocol for the collection of the participants data for the study. Written informed consent to participate in this study was provided by the participants’ legal guardian/next of kin.

## Author Contributions

AS and SM contributed to this study by initiating the project idea and design, writing the IRB proposal that was submitted and approved, and interpreting the results as they apply to clinical medicine. KC provided the clinical consultation. LA completed the statistical analysis, Texas Tech University Health Science Center El Paso, division of biostatistics and epidemiology. BG conceptualized, analyzed, writing, and editing of the manuscript. ER, JVM, VDP, and SI revised and edited the work critically for important intellectual content and final approval of the version to be published. All authors contributed to the article and approved the submitted version.

## Conflict of Interest

The authors declare that the research was conducted in the absence of any commercial or financial relationships that could be construed as a potential conflict of interest.

## Publisher’s Note

All claims expressed in this article are solely those of the authors and do not necessarily represent those of their affiliated organizations, or those of the publisher, the editors and the reviewers. Any product that may be evaluated in this article, or claim that may be made by its manufacturer, is not guaranteed or endorsed by the publisher.

## Abbreviations

ADHD: attention deficit hyperactivity disorder
BMI: body mass index
SES: socioeconomic status
DSM-V: diagnostic and statistical manual of mental disorders-5.
